# Vertebral body compression fracture after percutaneous pedicle screw removal in a young man

**DOI:** 10.1007/s10195-014-0328-5

**Published:** 2014-11-23

**Authors:** M. Cappuccio, F. De Iure, L. Amendola, A. Martucci

**Affiliations:** Department of Orthopedics and Traumatology - Spine Surgery, Maggiore Hospital, Largo Nigrisoli, 2, 40100 Bologna, Italy

**Keywords:** Complication, Compression fracture, Implant removal, Spine surgery

## Abstract

Hazards and potential complications associated with pedicle screw insertions have been reported. In contrast, complications due to implant removal are rarely described. An unreported case of acute vertebral body compression fracture following pedicle screw removal in a young man occurred during an episode of forceful coughing. Spinal implants need to be removed in cases of complications, pain or tissue irritation, and removal is mandatory when fixation involves L2 or the lower segments. Complications associated with spinal implant removal are rare but possible, and patients must be informed of this potential risk.

## Introduction

In thoracolumbar fractures, the choice between conservative or surgical treatment and the final outcome depend on several factors: type of fracture, presence of neurological impairment, the patient’s general condition and comorbidities, and associated injuries [1, 2].

Conservative treatment, especially bed rest, is not advisable in polytrauma patients, or in cases of claustrophobia, psychological disease, venous disease or previous deep venous thrombosis, obesity and bronchopulmonary diseases. In these situations percutaneous minimally invasive surgery can be an option [3]. This technique is used by the authors whenever conservative treatment is not advisable or when posterior open arthrodesis could represent an overtreatment.

Implant removal after percutaneous stabilization should restore a “normal” spine, provided screws are placed without damaging the facet joints, but this beneficial aspect has yet to be demonstrated. We report a case of acute vertebral body compression fracture of L1 following removal of pedicle screws previously placed to treat L2 fracture by means of percutaneous fixation. To the best of our knowledge, this type of fracture in a young man has not previously been reported.

## Case report

**Fig. 1:**
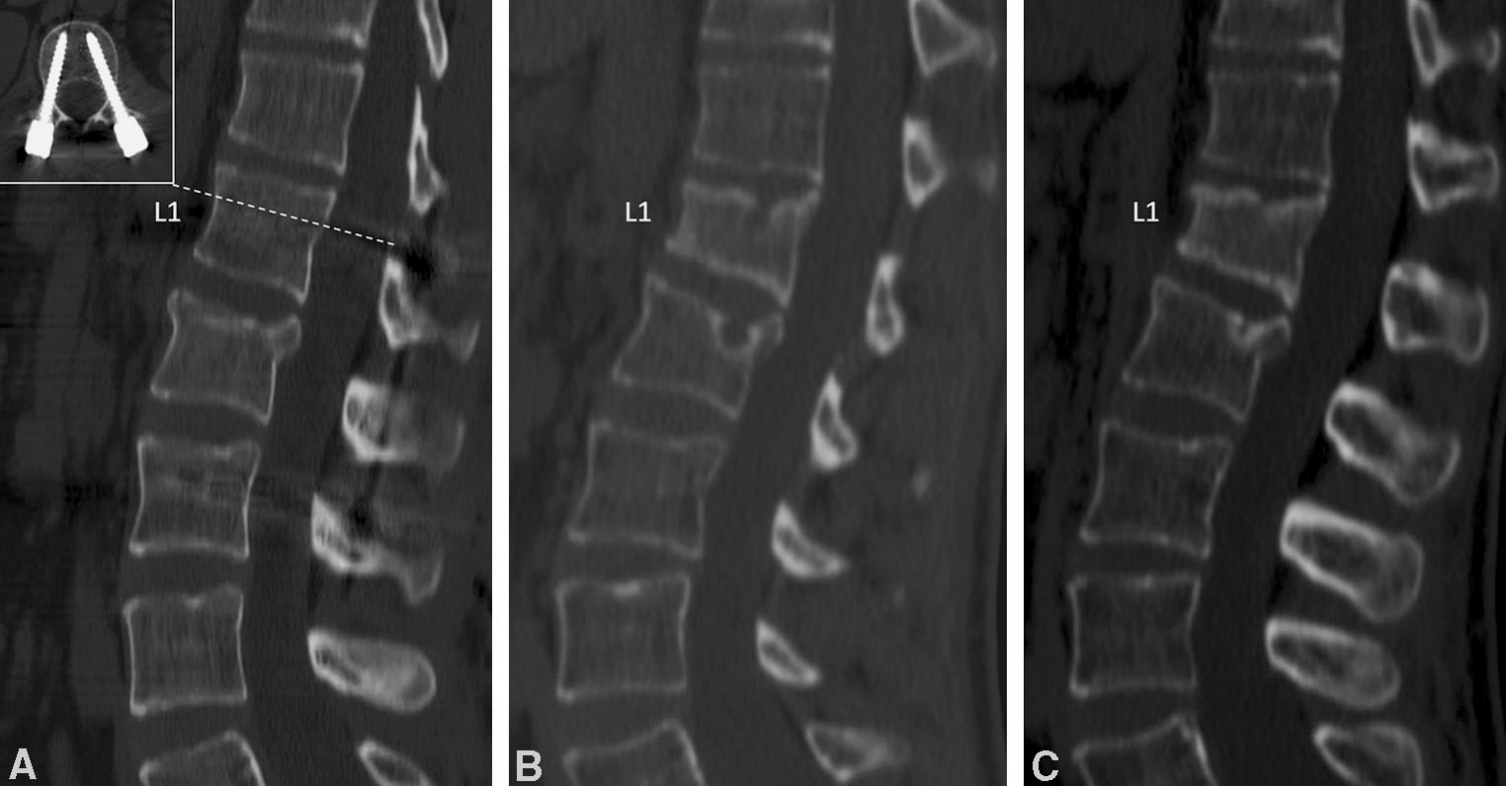
**a** Postoperative CT scan showed a good reduction of the fracture with correct positioning of pedicle screw in L1 (in the *box*). **b** One month after implant removal, a CT scan showed an acute wedge fracture on L1. **c** Fracture healing after conservative treatment with 3-point brace for 3 months

A 29-year-old man was treated by percutaneous stabilization from L1 to L3 (pedicle screw diameter: 5.5 mm in L1 and 6.5 mm in L3) for a L2 burst fracture (type A3.1 according to Magerl’s classification [1]) caused by a car accident (Fig. 1a).

After 6 months of follow-up the fracture was healed but the patient reported local pain, presumably due to mechanical irritation. Nine months after the first surgery, the spinal fixation was removed. No sign of implant failure or loosening was found on preoperative radiographs and on the operative field.

There were no intraoperative complications. The patient began to walk on the first post-operative day.

One month after the second surgery, the patient, suffering from bronchopneumonia, experienced a sharp pain in the upper lumbar spine during an episode of forceful coughing.

X-ray, computed tomography (CT) scan and magnetic resonance imaging demonstrated an acute wedge fracture (A1.2) of the superior plate on L1 (Fig. 1b). No signs of spondylodiscitis were present.

Very precise peripheral measurements by quantitative CT for the evaluation of bone density showed normal cortical and cancellous bone density. The patient had never used steroids. Body mass index was below 25. Laboratory tests were in the normal range except for markers of inflammation (WBC and ESR). C-reactive protein was normal.

The fracture was treated conservatively by a 3-point brace and analgesics for 3 months and healed without further progression of the kyphotic deformity (Fig. 1c). The patient returned to his previous job after 4 months. At the time of last follow-up, 12 months after the second surgery, he was completely pain-free.

## Discussion

In the management of thoracolumbar fractures, percutaneous pedicle screw fixation is used to reduce the approach-related morbidity of the open technique: iatrogenic muscle denervation, increased intramuscular pressures, ischemia, pain and functional impairment [4].

Hazards and potential complications associated with pedicle screw insertions have been reported [5].

In contrast, complications due to implant removal are rarely described. Vanichkachorn et al. [6] reported one case of potential large vessel injury during the removal of a broken pedicle screw. Waelchli et al. [7] showed two cases of acute vertebral compression fractures of the instrumented vertebral body adjacent to the fractured vertebra due to removal of pedicle screws. Both cases involved females who suffered from general osteoporosis and who had been previously treated for vertebral lumbar burst fracture. The authors assumed that the subcortical bone defect of the screw tracks was an important factor contributing to the additional weakening of the osteoporotic vertebral bodies.

To our knowledge, we describe the first acute vertebral compression fracture after percutaneous pedicle screw implant removal in a young man which occurred during an episode of forceful coughing.

Only one case of herniated lumbar disc associated with whooping cough is reported, probably as a result of the pressure effect from the tremendous force produced in the thoracic and abdominal cavity during a coughing access [8]. Vertebral fractures are reported in some cases associated with seizures in patients suffering from epilepsy [9, 10]. The authors assume that the forces generated during a tonic–clonic seizure can result in axial skeletal trauma, including thoracic or lumbar burst fractures. In the same way, the contraction force developed during a coughing access, although quantitatively lower, could result in a vertebral fracture if there is a subcortical bone defect of the screw tracks.

In the treatment of thoracolumbar fractures, percutaneous pedicle screw stabilization seems to be a good option.

Spinal implants need to be removed in cases of complications, pain or tissue irritations, and removal is mandatory when fixation involves L2 or the lower segments.

Complications associated with spinal implant removal are rare but possible, and patients must be informed of this potential risk.
